# Long-Term Oral Administration of Hyperimmune Egg-Based IgY-Rich Formulations Induces Mucosal Immune Response and Systemic Increases of Cytokines Involved in Th2- and Th17-Type Immune Responses in C57BL/6 Mice

**DOI:** 10.3390/ijms25168701

**Published:** 2024-08-09

**Authors:** Valentin Nastasa, Bogdan Minea, Aurelian-Sorin Pasca, Andra-Cristina Bostanaru-Iliescu, Alina-Elena Stefan, Daniela Gologan, Robert Capota, Liliana-Georgeta Foia, Mihai Mares

**Affiliations:** 1 Laboratory of Antimicrobial Chemotherapy, Faculty of Veterinary Medicine, “Ion Ionescu de la Brad” Iasi University of Life Sciences (IULS), 8 Mihail Sadoveanu Alley, 700489 Iasi, Romania; valentin.nastasa@iuls.ro (V.N.); sorin.pasca@iuls.ro (A.-S.P.); andra.iliescu@iuls.ro (A.-C.B.-I.); robert.capota@iuls.ro (R.C.); mihai.mares@iuls.ro (M.M.); 2 Department of Surgery, Faculty of Dental Medicine, “Grigore T. Popa” University of Medicine and Pharmacy of Iasi, 16 Universitatii Street, 700115 Iasi, Romania; georgeta.foia@umfiasi.ro; 3 Doctoral School, Department of Pathology, Faculty of Veterinary Medicine, University of Agronomic Sciences and Veterinary Medicine, 59 Mărăşti Boulevard, 011464 Bucharest, Romania; alina_stefan5@yahoo.com; 4 Department of Research and Development, Themis Pathology SRL, 56F 1 Decembrie 1918 Boulevard, 032468 Bucharest, Romania; daniela.gologan@yahoo.com; 5 Doctoral School, Department of Organic Chemistry, Faculty of Chemical Engineering and Biotechnologies, Politehnica University, 313 Splaiul Independenţei, 060042 Bucharest, Romania

**Keywords:** immunoglobulin Y, hyperimmune egg, oral administration, cytokines, interleukins, immune response, histopathology, hematology, biochemistry, C57BL/6 mice

## Abstract

Three hyperimmune egg-based formulations rich in immunoglobulin Y (IgY) were orally administered (daily, for up to 90 days) to C57BL/6 mice that were not microbially challenged. The serum levels of 32 cytokines were quantified every 30 days. Histopathology, hematology, and serum biochemistry investigations were also performed. As a sign of increased immune activity, lymphohistiocytic infiltrates were detected in the digestive tract and the liver after 30, 60, and 90 days of treatment. These infiltrates were also present in the lungs after 30 and 60 days, but not at 90 days. Blood analysis indicated systemic inflammation after 30 days of treatment: increases in pro-inflammatory cytokines, glycemia, total serum proteins, ALT, and ALP. After 60 and 90 days of treatment, the analyzed blood parameters showed mixed signs of both increased and decreased inflammation. The increased cytokines, which varied with formulation and time of exposure, indicated a combination of mostly Th17- and Th2-type immune responses. As the mice were healthy and housed in standardized sanitary conditions, and were not microbially challenged, the data were consistent with an interaction of IgY with the gut-associated lymphoid tissue as the main mechanism of action. This interaction generated a local immune response, which subsequently induced a systemic response.

## 1. Introduction

Hyperimmune eggs are produced by hens, which are repeatedly vaccinated with various antigens. The resulting antibodies are concentrated in the eggs, which can then be used for various therapeutic purposes [1]. Research on the subject demonstrated the efficacy of several formulations based on hyperimmune eggs in treating various conditions [2]. This product is therapeutically and economically convenient as it can contain a high amount of antibodies—up to 20 times more than colostrum [3,4]. Oral administration of these eggs with a high antibody titer was reported to provide protection against the specific pathogens used in the vaccine [5]. Hyperimmune eggs are a good source of antibodies for passive immunotherapy, which can be used successfully in the treatment of infectious diseases and can be an alternative to treatment with antibiotics [6,7,8]. The immune components of the hyperimmune egg can inhibit the growth and adhesion of bacteria to the intestinal walls and even inhibit their multiplication [9]. Obtained relatively easily and cheaply, these antibodies are known as immunoglobulin Y (IgY) and are used today to treat and prevent various diseases in humans and animals. They are reported to be very effective against *Escherichia coli*, *Salmonella* sp., rotaviruses, coronaviruses, transmissible gastroenteritis virus, and epidemic diarrhea virus [10,11,12,13,14]. The avian IgY is considered to be homologous with the mammalian IgG [2].

In the scientific literature, the proposed mechanisms of action for orally administered IgY are phenomena such as agglutination, adherence blockade, opsonization, or neutralization of toxins. In other words, in these investigations, the proposed mechanisms of action of IgY involve their binding to antigens found on various pathogens or toxins from the intestinal lumen. Therefore, most in vivo studies regarding orally administered IgY focused on their use as antimicrobials or involved their administration in contexts with natural or experimentally induced microbial or antigen challenges [2,10,15,16]. In these contexts, high doses seem to be more effective [17].

In 1988, Kulham et al. advanced the idea of the existence in the hyperimmune egg of certain cofactors with immunoregulatory roles that can influence the cytokines involved in the regulation of the inflammatory process [3]. Along with the antibiotic and anti-toxin activities of oral IgY, certain other studies also reported immunomodulatory activity, but even when this aspect was investigated, similar microbial challenge-type contexts were often used [15,18,19,20]. A mechanism of action for these immunomodulatory effects was either not proposed or was attributed to a positive interaction of IgY with the gut microbiome [19]. The beneficial effects of IgY on the gut microbiome were reported to stem from reducing the load of microbial pathogens, which would favor the presence of beneficial microbes [19,21,22]. Oral administration of IgY can alleviate clinical symptoms of intestinal infections and reduce the local inflammatory process by inhibiting various proinflammatory cytokines (IFN-γ, IL-1β, and TNFα) and overexpressing certain anti-inflammatory cytokines (IL-10) [17,20]. The immunomodulatory properties are also supported by the effects of hyperimmune egg administration in patients with arthritis (rheumatoid arthritis, osteoarthritis, and psoriatic arthritis), which led to a significant relief of symptoms even 30 days after cessation of administration [23]. Some authors suggested that, in fact, the immunomodulatory effects are conferred by certain IgY-associated peptides, which have a biological activity similar to that of the IgG-associated proline-rich polypeptide complex found in mammalian colostrum. Presumably, IgY would act as carriers for these peptides that can stimulate the production of IL-1β, TNFα, and nitric oxide [24,25,26].

Despite the evidence that orally administered IgY significantly influences the immune system, studies on their effects in long-term, high-dose administration are very scarce. Moreover, there are various IgY formulations on the market that are sold as food supplements for human use, for which no usage limits are specified.

The main objective of the present study was to assess the response in C57BL/6 mice to long-term (up to 90 days) oral administration of three IgY-containing formulations—fresh (HE) and freeze-dried hyperimmune egg (fdHE)—and an immunoglobulin Y extract (Yext) in a context without a microbial challenge where the corresponding antigens of these IgY were either absent or not present in significant amounts in the digestive tracts of the animals. Our results show the development of a local and systemic immune response and also provide a certain amount of evidence to support certain mechanisms of action of orally administered IgY that do not involve their binding to their antigens.

## 2. Results

### 2.1. Clinical Observations

**Figure 1:**
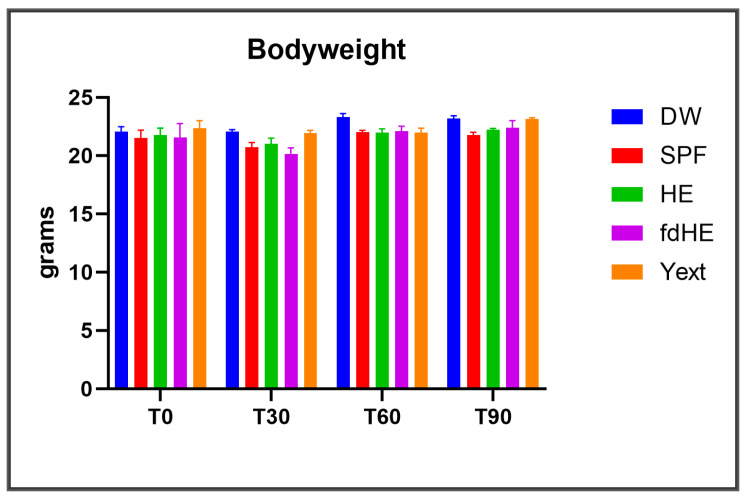
Bodyweight (mean ± SD) in the five experimental groups at the beginning of the experiment (T0) and at the three harvesting time points of 30 days (T30), 60 days (T60), 90 days (T90). The five groups received distilled water (DW), specific pathogen-free (SPF) egg, fresh hyperimmune egg (HE), freeze-dried hyperimmune egg (fdHE), or an IgY extract (Yext).

In addition to the three treatment groups that received the above-mentioned IgY-rich formulations (HE, fdHE, and Yext), this study also included two control groups that received distilled water (DW) and a specific pathogen-free (SPF) egg emulsion. No behavioral changes were recorded during this study. The mice from the five groups showed normal daily activity, with no episodes of apathy, vomiting, or diarrhea. In general, the mice from the groups that received egg-containing products (SPF, HE, and fdHE) had slightly lower body weights compared with the DW control and the Yext group (Figure 1, Supplementary Table S1). The differences were maximal after 30 days of treatment.

### 2.2. Histopathology Results

The macroscopic assessment of the appearance of the tissues showed no changes in volume, color, shape, consistency, surface area, or cross-sectional area. The histological examination revealed certain changes in the liver, duodenum, colon, spleen, kidneys, and lungs.

**Figure 2:**
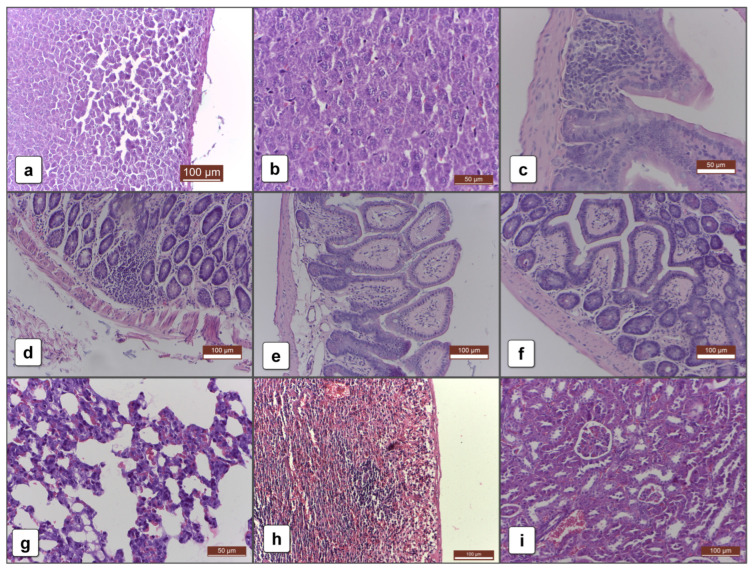
Histopathology aspects: (**a**) T30 Liver—lymphohistiocytic infiltrate; (**b**) T90 Liver—lymphohistiocytic infiltrate; (**c**) T30 Duodenum—lymphohistiocytic infiltrate; (**d**) T60 Duodenum—lymphohistiocytic infiltrate with eosinophils; (**e**) T90 Duodenum—lymphohistiocytic infiltrate with amyloidosis; (**f**) T90 Colon—lymphohistiocytic infiltrate with amyloidosis; (**g**) T90 Lung—without lymphohistiocytic infiltrate; (**h**) T30 Spleen—congestion caused by anesthesia and formaldehyde infusion; (**i**) T90 Kidney—without lymphohistiocytic infiltrate.

After 30 days of administration of products containing IgY, no life-threatening changes were found in the liver. A reduced lymphohistiocytic proliferation, however, was present in groups fdHE and Yext (Figure 2a), indicating a local immune response. At 60 days, this lymphohistiocytic infiltrate was noted in mice from groups SPF and HE too. Sporadically, mild steatosis was observed in all groups. After 90 days, the histological picture of the liver was dominated by the lymphohistiocytic infiltrate in the groups treated with IgY-containing products—HE, fdHE, and Yext (Figure 2b). Therefore, at 90 days, the normal egg components appeared to elicit a reduced immune response in the liver compared with IgY.

Regarding the digestive tract, a reduced lymphohistiocytic infiltrate was noted in the duodenum and colon in groups SPF, HE, and fdHE at 30 days (Figure 2c). The same immune activity, but with a higher intensity, was noted in the groups treated with IgY-containing products (HE, fdHE, and Yext) after 60 days (Figure 2d). The intensity was even higher at 90 days, and it was associated with amyloidosis in the duodenum (Figure 2e), in the fdHE group, or in the colon in groups SPF, HE, fdHE, and Yext (Figure 2f). This high immune activity at 90 days was consistent with that in the liver, but in the digestive tract, the normal egg components appeared to continue to elicit a local immune response.

The histological investigations performed on the lungs showed in groups SPF, HE, fdHE, and Yext an interstitial lymphohistiocytic proliferation at 30 and 60 days, which was no longer present at 90 days (Figure 2g). This indicated that the local immune response at the level of the digestive tract was accompanied by an increase in systemic immune activity, which manifested itself particularly at the level of the lungs. Congestion was also encountered, but without clinical significance, as it is a known effect of anesthesia and formaldehyde infusion. This congestion was also present in the spleen (Figure 2h). In group HE, at 90 days, a slight lymphoid hyperplasia in the lymphoid follicles was also observed, which indicated a certain level of immune activity.

In the kidney, swelling in the proximal portion of the urinary tubules’ epithelium was observed in all groups at 30, 60, and 90 days. The high protein intake or a certain level of systemic immune activation induced by the administered formulations are possible causes for this swelling, but its presence in the control group that was administered only water suggests that other factors may have played a role, such as the daily stress of gavage. Other indicators of an inflammatory process were not noted (Figure 2i).

No skin changes were recorded throughout the experiment.

### 2.3. Serum Cytokines

**Figure 3:**
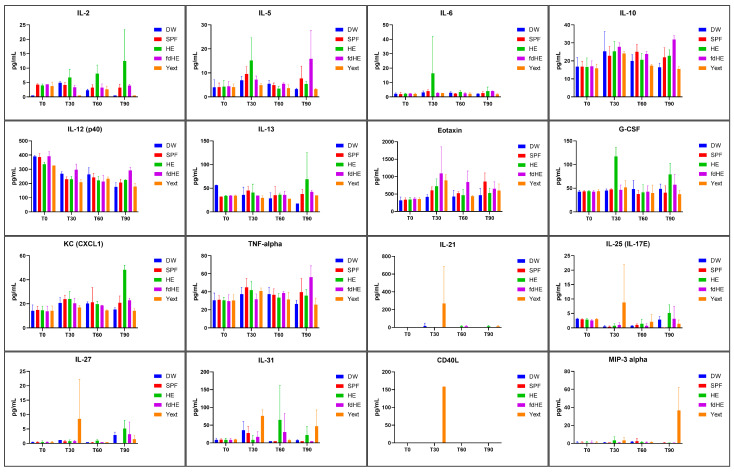
Serum levels (mean ± SD) of the cytokines that were increased in the treatment groups (HE, fdHE, Yext) compared with both control groups (DW, SPF): at the beginning of the experiment (T0), after 30 days (T30), 60 days (T60), and 90 days of treatment (T90).

Cytokines and chemokines varied differently between groups over the course of the 90-day study. Table 1 shows the cytokines that were significantly increased in the IgY-treated groups compared with both control groups (DW and SPF), along with the *p* values for each comparison. Table 1 also presents the *p* values for the significant differences between the sampling times. The serum levels of the increased cytokines are presented in Figure 3, while the serum levels of all 32 measured cytokines can be found in Table S2.

Compared with the two control groups (DW and SPF), at 30 days, IL-6 and G-CSF showed a significantly increased serum level after fresh hyperimmune egg administration. IL-5 was increased compared with the DW group (*p* = 0.0293) but not to the SPF group (*p* = 0.2566). The fdHE-treated group had Eotaxin significantly elevated. The Yext group had the most cytokines that were increased after 30 days of treatment, i.e., IL-21, IL-25 (IL-17E), IL-27, and CD40L. IL-3 (*p* = 0.0139), IL-17A (*p* = 0.0014), and Eotaxin (*p* = 0.0379) were also significantly increased compared with the DW group but not compared with the SPF group (*p* = 0.0796, *p* = 0.2782, and *p* = 0.3866, respectively). IL-17F (*p* = 0.0078) and IL-23 (*p* = 0.0026) increased compared with the SPF control group but not the DW group (*p* = 0.0942 and *p* = 0.0663, respectively).

After 60 days of treatment, the cytokines increased, and after 30 days, they dropped back to baseline levels. In fact, in the treated groups, most cytokines had levels that were very similar or not significantly different from those in the control groups, with the exception of IL-31, which had significantly higher values in the group that received fresh HE.

After 90 days, IL-2, IL-13, G-CSF, and KC (CXCL1) were significantly increased in the HE-treated group. The fdHE-treated group had increased levels of IL-5, IL-10, IL-12 (p40), and TNFα. IL-4, IL-9, IL-17A, and IFN-γ increased significantly compared with the DW group but not compared with the SPF group. In both groups (HE and fdHE), GM-CSF increased significantly compared with the DW group but not compared with the SPF group. In the group that received the IgY extract, only MIP-3α was significantly elevated at the end of the 90 days.

Most cytokines that were increased at 30 and 90 days, compared with the control groups, also had elevated levels when compared with T0 and T60 levels within the same group, supporting a general picture of cytokine increases after 30 and 90 days of treatment but not after 60 days.

The cytokines that were increased compared with both control groups were indicative of an immune response elicited by the IgY or their fragments. The cytokines that were increased compared with the DW group but not compared with the SPF group suggested that the normal egg components also elicited an immune response. In the cases where cytokines were increased compared with the SPF group but not the DW group, the cause seemed to be the relatively small sample size and the inter-individual variability.

Table 2 displays the types of immune responses in which these increased cytokines are reported to play an active role, together with a few references. Most of the increased cytokines in our experiment were reported to be involved in Th2 and Th17-type immune responses. The Th2 response suggested the presence of allergic-type processes, while the Th17 response was indicative of the activation of mucosal defense mechanisms through the contact of the digestive tract mucosa with IgY or IgY fragments.

### 2.4. Serum Biochemistry

**Figure 4:**
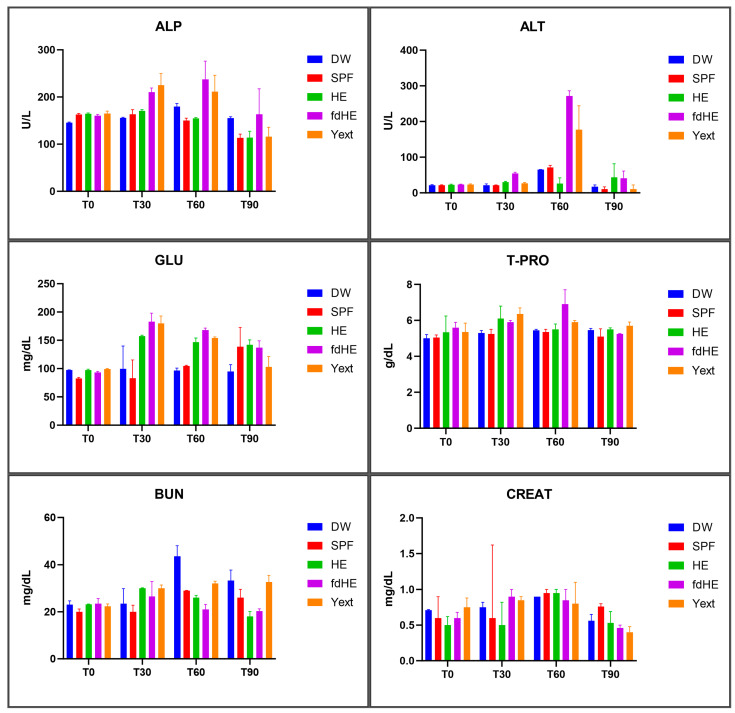
The levels of serum biochemistry parameters (mean ± SD): alkaline phosphatase (ALP), alanine aminotransferase (ALT), glucose (GLU), total proteins (T-PRO), blood urea nitrogen (BUN), and creatinine (CREAT): at the beginning of the experiment (T0) and at the three harvesting time points of 30 days (T30), 60 days (T60), 90 days (T90).

The results of the serum biochemistry investigations are presented in Figure 4 and in Table S3. Creatinine levels did not show significant differences between groups throughout the experiment.

After 30 days of treatment, all treated groups had increased levels of GLU compared with the control groups. In addition, the Yext group had increased levels of T-PRO, BUN, and ALP; the HE group had increased BUN and T-PRO; and the fdHE group had increased ALP values. This was suggestive of a certain level of systemic inflammation and, in the case of the fdHE and Yext groups, some hepatic stress.

At 60 days, GLU levels remained significantly higher in the treated groups. In the HE group, all the other parameters decreased to levels that were not different from the control groups or, in the case of ALT, to significantly decreased levels. The fdHE group also had increased levels of ALP, T-PRO, and particularly ALT. The Yext group had similar increases, but only the ones in ALT were statistically significant. This indicated the persistence of the inflammation and, for groups fdHE and Yext, of the hepatic stress.

At 90 days, the HE group had decreased levels of BUN, compared with both control groups, and decreased ALP and increased GLU, compared with the DW control. The fdHE group had higher levels of GLU, compared with the DW control, and increased ALP, compared with the SPF control. The Yext group had higher BUN levels but lower GLU levels compared with the SPF group and lower ALP levels compared with the DW group. These results suggested that some level of inflammation was still present at 90 days, but the hepatic stress seemed to have subsided in groups fdHE and Yext.

### 2.5. Hematology

Regarding the parameters of RBCs and platelets (Table 3), the treated groups were most often either not significantly different or they were different compared with one of the control groups but not compared with both.

At the beginning of the experiment, prior to any treatment, the HE group had lower HGB values compared with the SPF control (*p* = 0.0002). After 30 days of treatment, the HE group had lower values for HGB (*p* = 0.0086) and HCT (*p* = 0.0388) compared with the SPF group. The HGB, however, increased in group HE at every sampling time compared with the previous one. The Yext group had higher HGB values compared with the DW control (*p* = 0.0086).

At the end of 60 days of treatment, the fdHE-treated group had increased RDWc (*p* < 0.0001 vs. DW; *p* = 0.0018 vs. SPF) and RDWs (*p* = 0.0002 vs. DW; *p* < 0.0001 vs. SPF) values compared with both control groups. An elevated RDW is indicative of inflammation, hyperglycemia, and hepatic stress.

After 90 days of treatment, the DW control group had lower HGB (*p* < 0.0001 vs. SPF; *p* = 0.0002 vs. HE; *p* < 0.0001 vs. fdHE; *p* = 0.0046 vs. Yext) and HCT (*p* = 0.0044 vs. SPF; *p* = 0.0006 vs. HE; *p* < 0.0001 vs. fdHE; *p* = 0.0002 vs. Yext) values compared with the SPF control and all treated groups. The SPF group had higher MCHC values compared with all the other groups (*p* = 0.0009 vs. DW; *p* = 0.0001 vs. HE; *p* < 0.0001 vs. fdHE; *p* < 0.0001 vs. Yext), but only the Yext group had lower MCHC values compared with both controls (*p* = 0.0326 vs. DW; *p* < 0.0001 vs. SPF).

Given the differences at T0 and T30 and the pattern and amplitude of the variation, HGB, HCT, MCHC, and RDW appeared to be influenced by an interplay between individual variability, the high nutritional value of the egg, the adaptation to this new food, and the presence of inflammation.

The values for RBC, MCV, and MCH did not show any statistically significant differences. 

Concerning the platelets, at 30 days of treatment, the fdHE group had higher MPV values compared with both controls (*p* = 0.0099 vs. DW; *p* = 0.0009 vs. SPF) and higher PLT values compared only to the DW control (*p* = 0.0006 vs. DW). There were no significant differences at 60 days. At 90 days, the two control groups were divergent, being at the two extremes, with the treated groups between them. The SPF group had the highest values for PLT and PCT and the lowest values for MPV, PDWc, and PDWs, while the situation was opposite for the DW group. The differences between the two were statistically significant for the PLT, PDWc, and PDWs values. The HE group had significantly higher PLT values compared with the DW control and higher PDW values compared with the SPF group. The fdHE group had lower PLT values and higher PDWc and PDWs values compared with the SPF control. The Yext group had higher PLT values and lower PDWc and PDWs values compared with the DW group.

Similarly to the red blood cell parameters, the platelet parameters appeared to have been influenced by an interplay between individual variability, normal time fluctuation, and the presence of inflammation.

**Figure 5:**
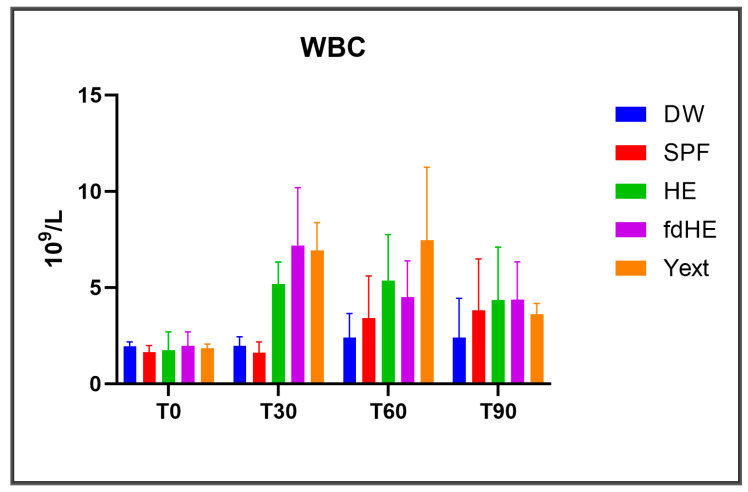
Total WBC levels (mean ± SD): at the beginning of the experiment (T0) and at the three harvesting time points of 30 days (T30), 60 days (T60), 90 days (T90).

**Figure 6:**
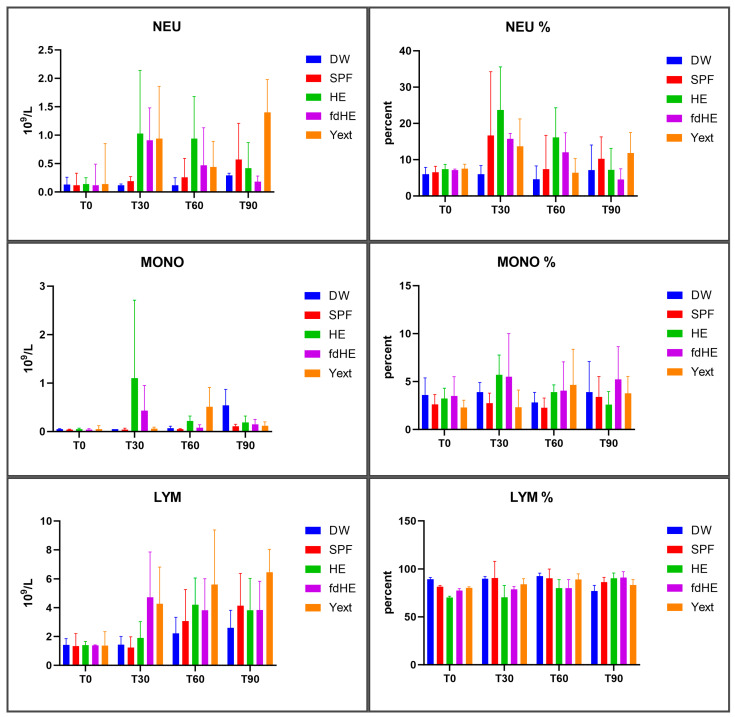
The levels of neutrophils (NEU), monocytes (MONO), and lymphocytes (LYM) in absolute numbers (10^9^/L) and percentages of total white blood cells (mean ± SD) at the beginning of the experiment (T0) and at the three harvesting time points of 30 days (T30), 60 days (T60), 90 days (T90).

The results regarding white blood cells are presented in Figure 5 and Figure 6, and in Table S4.

As absolute numbers, all treated groups had more white blood cells in general (Figure 5) and specifically more neutrophils and monocytes (Figure 6) compared with the control groups after 30 and 60 days of treatment, which signaled increased systemic immune activity. After 90 days, the situation was different, depending on the treatment and the type of white blood cell considered. The HE group had a maximum of monocytes and neutrophils at 30 days, which then decreased at 60 and 90 days. The lymphocytes slightly increased at 30 days, peaked at 60 days, and then slightly decreased at 90 days. The differences, however, were statistically significant only for monocytes at 30 days, which increased compared with both control groups. The fdHE group had a maximum of white blood cells at 30 days, followed by a decrease at 60 days. At 90 days, neutrophils further decreased, total WBCs and lymphocytes remained the same, and monocytes increased slightly. The differences, however, were not statistically significant, except for total WBCs at 30 days, which increased compared with both control groups. In the Yext group, total WBCs significantly increased at 30 days; they peaked at 60 days and decreased at 90 days to levels that were similar to those of control groups. Every type of WBC, however, behaved differently. Monocytes were unchanged at 30 days, peaked at 60 days (*p* > 0.05), and then decreased at 90 days; lymphocytes increased up to the end of the experiment, but the differences were significant only at 90 days compared with the DW control. Neutrophils had an increase at 30 days, followed by a decrease at 60 days, and then an even higher increase at 90 days, but the differences were significant only at 90 days compared with the DW control.

Eosinophils and basophils in the blood were below the detection limit of the analyzer for all groups throughout the experiment.

Although not statistically significant, the differences in the parameters pertaining to white blood cells were in agreement with those concerning the other blood parameters analyzed. They were consistent with increased systemic immune activity at 30 days (also supported by increased cytokines, glycemia, serum proteins, and ALP), while at 60 and 90 days there were mixed signals of both elevated and decreased immune activity.

## 3. Discussion

In most published research on orally administered IgY, they are used as anti-microbials or anti-toxins, implying mechanisms of action involving the binding of these immunoglobulins to their antigens (challenge-type contexts). Even the formulations used in our experiment were initially designed for use as antimicrobials. That is why they contained IgY against common human pathogens, which are not significant components of either the human or murine gut microbiomes [45]. If present in significant (infective) amounts in the digestive tract, these species would have caused evident digestive distress. In our study, the mice were housed in standardized sanitary conditions; they were not challenged with these microbes, nor did they exhibit vomiting, diarrhea, or other signs of altered digestion, including the DW control group, which only received distilled water. It is, therefore, reasonable to assume that these pathogens were not present in significant amounts in the digestive tract of the mice. Thus, in our experiment, the IgY were administered in a non-challenge context, where their target antigens were either absent or not present in significant amounts in the intestinal lumen. This type of research where IgY is administered in a non-challenge context, particularly in long-term setups, is much less present in the literature. Although a certain level of interaction with the gut microbiome is not excluded, it does not appear to be the main mechanism of action. Most research reports beneficial effects of the interaction of IgY with the gut microbiome, particularly a reduction of inflammation [17,22]. In our experiment, the initial effects of the orally administered IgY were clearly proinflammatory. In these cases, the IgY themselves appear to function as antigens that interact with the gut-associated lymphoid tissue (GALT) and the immune components of the liver.

Proteins or digested protein fragments from the intestinal lumen are constantly being sampled, usually by enterocytes or specialized cells (e.g., M cells, goblet cells) located in the intestinal and colonic mucosae. These protein samples are subsequently taken up by cells capable of presenting antigens, such as intestinal dendritic cells and macrophages, but also the sinusoidal epithelial cells, Kuppfer cells, and dendritic cells in the liver [46,47,48,49], or can sometimes be passed on directly to B cells [50]. Depending on factors like molecular structure, dosage regimen, sampling location, local immune status, etc., the proteins that reach the GALT can induce immune responses in the form of either oral immune tolerance or inflammation [46,49,50,51].

In our study, the presence of the lymphohistiocytic infiltrate at the level of the digestive tract (small intestine, colon) indicated a local immune activation probably induced by the interaction of IgY or fragments of IgY with GALT. Immunoglobulins Y were reported to resist digestion, especially their Fab’ fragments [52,53]. Although susceptible to pepsin digestion, IgY are relatively resistant to trypsin and chymotrypsin [10], so the intact molecules that make it to the intestine have higher chances of being sampled into the GALT or absorbed into the bloodstream. Indeed, IgY was found intact in the blood after intragastric administration. The absorbed quantities were highest in very young mammals and decreased with the advancement of age [16,54]. Moreover, IgY (particularly from chickens) was reported to be a more effective trigger of mammalian immunity compared with IgG [55], and the Fab’ and pFc’ fragments were reported to induce a stronger immune response than the intact IgY [56]. In our investigations, the presence of the lymphohistiocytic infiltrate in the liver is consistent with these reports; a crossing of the intestinal barrier and entrance in the portal circulation of IgY or their fragments is a plausible explanation for this infiltrate. The presence of the same type of lymphohistiocytic infiltrate at the level of the lungs was an indication that the local mucosal immune activation had extended systemically.

The hematology and blood biochemistry data, as well as the analyzed cytokines, were consistent with the histopathology data, supporting both the occurrence of the local intestinal and hepatic inflammation and the systemic spread.

Elevated RDW is reported to be caused by inflammation, liver injury, and increased HbA1c following hyperglycemia [57,58,59,60]. ALT and ALP are biochemical parameters that are frequently used to assess liver function and health, and their increased levels are associated with liver inflammation [61]. Moreover, intestinal ALP was reported to be part of the innate immune response and have anti-inflammatory activity [62], as did tissue non-specific ALP [63]. In our experiments, the freeze-dried hyperimmune egg seemed to have had a more pronounced effect on the liver, as indicated by the raised RDWc, RDWs, ALT, T-PRO, and ALP at 60 days in the group treated with this product. The raised MPV at 30 days might have been an early sign of this hepatic stimulation, as this parameter was reported to be associated with inflammation in general [57], but also specifically with liver inflammation [64,65]. The IgY extract also seemed to induce some hepatic stress, although slightly less, as it only increased ALT and ALP. The biochemical parameters showed the fresh hyperimmune egg to have induced the least stress on the liver.

Increased glycemia is an important symptom of inflammation [66], as the pro-inflammatory M1-type macrophages are glycolytic [67]. Total serum proteins can also be used as an inflammation marker, as they increase during inflammation because of increased production of acute-phase proteins and cytokines [68,69,70]. The slight increases in glycemia and total protein in the treated groups would indicate a persistent but moderate level of systemic inflammation. The fact that the inflammation is somewhat attenuated is also supported by the slightly increased RBC, as acute inflammation is reported to reduce RBC survival because of an increased production of reactive oxygen species [67]. The increased values of RBC and HGB are also an indication that the induced systemic inflammation did not cause iron sequestration. This withholding of iron is characteristic of the M1 macrophage phenotype, while the switch to the pro-resolving M2 phenotype is associated with iron release [71]. This could indicate the presence of pro-resolving/anti-inflammatory M2 macrophages and the co-existence of inflammation and resolution processes.

Most of the modified cytokines had increased levels, outlining a general pro-inflammatory picture. The nature of the increased cytokines suggested a mix of mostly Th2 [27,28,34,35,36,37,38,41,43] and Th17 responses [29,30,31,32,33,36,39,42,44]. Our data, therefore, would support the presence of a mucosal Th17 inflammatory response at the level of the gastro-intestinal tract mucosa, which was accompanied by a Th2 allergic-type systemic response. The gut itself may also have been a source of Th2 cytokines, as this type of immune response was reported to increase at this level after oral administration of protein antigens [49]. The cytokine levels in the treated groups showed the inflammatory response to have been more intense at 30 and/or 90 days and back to base-line or control group levels at 60 days. In a study performed on SPF pigs (i.e., in a context without microbial challenge) where the immunological activity of IgY was assessed based on the serum level of anti-IgY antibodies, the immune response to IgY administered to the intestinal mucosa appeared to often exhibit a similar pattern to the one in our experiments, that is, a maximum at 28 days followed by a decrease at around 60 days [72].

The elevated cytokines were different between groups at each collection time and between collection times within the same group. This could have been caused by chemical differences between the three IgY formulations that were available, but also by the phenomena of redundancy and pleiotropism, which are characteristic of cytokines [73]. This would also explain the interesting situation where the elevated cytokines were completely different between the HE and fdHE formulations, which, technically, had very similar contents.

The Yext group had more elevated cytokines at 30 days, which suggested that the immunological effects occurred earlier. Also, there were additional increased cytokines that did not reach statistical significance but were close, suggesting a potentially stronger activity at 30 days compared with the other tested IgY-based products. The formulations where IgY were part of the whole egg matrix (HE and fdHE) appeared to have a somewhat delayed action, with more elevated cytokines at 90 days. A possible explanation could be the presence of albumin in the formulations derived from whole eggs, which would reduce the pepsin-to-IgY ratio in the stomach. Such a reduction was reported to significantly extend the IgY digestion time [53], which would mean that in these groups, more intact IgY reached the intestinal mucosa. Intact IgY were reported to be less immunogenic than their Fab’ fragments [56]. As the digestive tract of the animals adapted to the high protein diet, the pepsin:IgY ratio increased, which raised the amount of the more immunogenic Fab’ fragments that reached the intestine. This could explain the delayed increase of cytokines at 90 days in the groups that received fresh or freeze-dried hyperimmune eggs.

MIP-3α is a chemokine that attracts lymphocytes and neutrophils and is particularly involved in the activity of mucosal lymphoid tissues [74,75]. The increased levels of MIP-3α in group Yext at 90 days were associated with increased levels of lymphocytes and neutrophils, which indicated a possible resurgence of inflammation caused by the continual administration of IgY.

Although cytokines were decreased at 60 days, the histopathology investigations showed the persistence of the lymphohistiocytic infiltrate, which was present throughout the experiment. Moreover, the hematology and biochemistry data also showed signs of inflammation at 60 days in the form of elevated blood levels of RDWc, RDWs, ALT, T-PRO, ALP, and glucose. The pattern of cytokine variation superposed on the histopathology, hematology, and biochemistry data would indicate a co-existence of this persistent moderate inflammation with systemically spread processes of inflammation resolution. Although they did not reach statistical significance, the variation patterns of neutrophils and monocytes also appeared to be consistent with this hypothesis.

Inflammation resolution is not a passive reduction of inflammation but a highly regulated process. Important steps in the resolution of inflammation are stopping the influx of neutrophils, reducing macrophages, and decreasing pro-inflammatory cytokines [76,77]. The reduction of neutrophils at 60 days in our study, as well as the decreased levels of pro-inflammatory cytokines, were consistent with the possibility of resolution processes taking place. Moreover, the presence of eosinophils in the lymphohistiocytic infiltrates can be interpreted as a sign of a Th2 response, but they can also indicate the presence of processes of resolution, as they are important players in inflammation resolution by producing significant amounts of pro-resolution mediators [77,78].

Usually, an innate inflammatory response is developed quickly and then resolved in a few days [79]. All the signs of moderate inflammation that were found seemed to point towards the presence of a persistent and moderately acute inflammatory process that was maintained by the daily administration of antigen rather than true chronic inflammation. The coexistence with processes of inflammation resolution could be explained by the repeated administration of IgY. Persistent stimulation was reported to induce monocytes with an anti-inflammatory phenotype [80]. Furthermore, the phagocytosis of neutrophils triggers a phenotype switch in macrophages, from pro-inflammatory to pro-resolution [81]. In addition to the repeated administration of antigen, another possible cause of the lymphohistiocytic infiltrates present at various levels would be the presence of long-lasting macrophages, a set of immune cells that persist at the sites of inflammation long after resolution [81].

The histopathological, hematological, and immunological analyses of the SPF group showed that normal egg components also induced an immune response. The egg is recognized as a functional food that contains a variety of bioactive compounds that can influence pro- and anti-inflammatory pathways. Compounds such as lutein, zeaxanthin, ovalbumin, ovotransferrin, ovomucin, lysozyme, or avidin can have immunomodulatory effects but can also trigger allergic responses [82,83,84,85]. The immunological effects of the normal egg components, however, appeared to be limited to the mucosae, without significant changes in the levels of blood markers.

The presence of a lymphohistiocytic infiltrate in both the digestive tract (administration site) and the lungs, similar to the IgY-treated groups, emphasized the immunological link that exists between these two mucosae. This is consistent with other reports of immunological connections between various mucosal sites [50,86]. A possible explanation would be the reverse migration of some neutrophils from the inflammation sites. On their way back to the bone marrow, the lungs appear to be an important stop [87,88].

The groups that received products containing the complete egg (SPF, HE, and fdHE) showed a decrease in bodyweight at 30 days, but the animals returned to their initial weight at 60 days. This may have been caused by a process of metabolic adaptation to a new food source as well as the high protein content of this new food, as protein is known to reduce appetite. The Yext group did not show a bodyweight decrease but rather a delay in bodyweight gain compared with the DW control, probably because of the lower protein intake and the early immunologic effects of isolated IgY, which showed a maximum at 30 days.

In oral immunotherapy, high short-term doses of orally administered antigens or low doses (either short-term or long-term) were reported to usually induce oral tolerance, a state where the immune response to the antigen is suppressed, through different mechanisms, including the generation of regulatory T cells (T_regs_) [46,49,89,90]. The suppression induced by oral tolerance does not remain locally confined but extends systemically, probably through the migration of some of the T_regs_ [46,51]. Without causing generalized immunosuppression, these T_regs_ can then act on other inflamed body sites in inflammations caused by other antigens that occur at the same time or in close proximity to the tolerance-inducing event. This phenomenon was called bystander suppression, a potentially important tool in oral immunotherapy, particularly in situations where the antigen causing a certain condition is not known [47,51,91,92,93,94,95].

Therefore, a reasonable hypothesis would be that, at low doses, one of the mechanisms through which IgY acts may be the induction of oral immunotolerance (possibly through the proliferation of T_regs_). Indeed, studies in which low doses of IgY were used report a lack of inflammation or even anti-inflammatory activity. The oral administration of IgY was reported to have an anti-inflammatory effect on the intestinal mucosa of mice with castor oil-induced diarrhea [96]. It also reduced the symptoms in a rat model of arthritis [97]. The oral administration of IgY in humans did not induce increases in blood inflammation markers [97]; considering the metabolic equivalency, the dose used in that study was significantly lower than the one used in our experiments. Oral IgY was also reported to significantly reduce muscle soreness caused by physical exertion as well as blood inflammation markers [98], which highlights the fact that the effects of orally administered IgY are not just locally limited to the intestinal mucosa but extend systemically.

Low doses of IgY were reported to reduce symptoms and markers of inflammation in a murine model of psoriasis [99,100]. Although the beneficial effects were attributed to interactions of IgY with the microbiome, in our opinion, the data also supported (or at least did not exclude) the contribution of oral immunotolerance, which may have acted on the psoriatic-like inflammation through bystander suppression.

In our study, high doses of long-term administered IgY-induced local inflammation appeared to subsequently induce systemic immune responses. A possible reason would be the location of the IgY contact with GALT. The stimulation of the lymph nodes associated with the proximal segments of the small intestine tends to generate immune tolerance, while the distal lymph nodes have more pro-inflammatory tendencies [101]. Because the IgY was administered in high doses, it is reasonable to conclude that significant quantities reached the posterior segments of the small intestine.

Another site of immune activation was the colon, as indicated by the lymphohistiocytic proliferation at this level. This would support the fact that enough IgY or Fab’ fragments reached the colon due to the high dose and repeated administration. Similar to the lymph nodes draining the distal small intestine, the ones associated with the colon are also reported to react in a predominantly pro-inflammatory manner [51]. Although the dendritic cells of the colon were reported to be less influenced by food-derived compounds and less prone to generating systemic immune responses [51,102], a protein antigen administered directly to the colon, i.e., ovalbumin, induced a stronger systemic immune response compared with oral administration, possibly due to the lower amount of proteases at this level and with the longer time that the antigen spends in this segment [103]. In our experiment, however, it was the IgY or their Fab’ fragments that appeared to be responsible, at least partially, for the immune activation of the colon, since ovalbumin was not administered directly to the colon but orally, and since the lymphohistiocytic infiltrate was also present in the group that received only the IgY extract. Furthermore, antigen stimulation in an already inflammatory context tends to induce more inflammation than oral tolerance [46,104]. This could explain the maintenance of moderate immune activation by repeated IgY administration.

Compared with our study, higher doses administered daily to Beagle dogs did not induce significant changes in the hematologic and biochemical parameters that could indicate inflammation, showing that it is not just the metabolic rate but various other features of digestion, physiology, and immunity that determine the effects of orally administered IgY [21]. This is consistent with the results of Torché et al., who reported difficulties in translating the results of mouse immunization for larger animals, such as pigs [72].

The results of our investigations seem to indicate that, in an environment without a pathogenic microbial challenge, IgY acts as an oral antigen delivery system. Although the gastric low pH and the partial digestion by pepsin seem to reduce the effectiveness of IgY as antibodies (e.g., antimicrobial or anti-toxin activity) [16], these would not reduce their effectiveness as a system that delivers non-specific antigens (i.e., Fab’ fragments or whole IgY) to GALT or in the bloodstream. As the Fab’ and pFc’ fragments are more immunogenic, pepsin digestion may actually be desirable when orally administered IgY are used as immunomodulators.

The systemic inflammation induced by the oral administration of IgY appeared to subsequently trigger and co-exist with systemic processes of inflammation resolution. Normally, the pro-inflammatory and resolution processes coexist without the need for repeated immune stimulation. In fact, the pro-inflammatory processes are the ones that trigger the resolution of the inflammation [105]. An interesting avenue for future research would be to investigate whether these processes of inflammation resolution can act in other inflamed body sites by resetting and restarting frustrated resolution processes, either through various chemical mediators or through cellular factors such as long-lived neutrophils or macrophages with a pro-resolving phenotype. Certain neutrophils have been reported to escape apoptosis and return to circulation, being able to act in other body sites [78,87,88]. Such a “bystander resolution” (so called by analogy with bystander suppression) would be a useful immunotherapy tool in chronic inflammations. Scientific literature that specifically examines such interplays between local and systemic inflammation and resolution of inflammation in mucosal immunology contexts is scarce [106], but strategies that involve inducing short-term local or systemic inflammation (such as resistance training or sauna) and subsequently reducing it in other body sites have become increasingly well documented [107,108,109,110].

IgY supplements appear not to be completely innocuous products that can be administered indefinitely and at any dose. Some commercial hyperimmune egg supplements do not communicate a precise dose, nor do they state any restrictions regarding treatment duration. Although the translation of mouse data into applications for larger mammals is not a straightforward process and the immune response may not exhibit the same intensity in larger mammals, our data and other reports suggest that more research and care should be put into establishing appropriate dosage regimens. Moreover, certain inflammation markers should be monitored during and after the treatment, and a certain level of caution should be used in general. With high doses, a potentially beneficial treatment strategy to be investigated would be periodical cessations of IgY administration to eliminate co-existent inflammation, which might improve the resolution of inflammation. Persistent inflammation increases the risk of frustrated resolution and subsequent chronic inflammation [81,111]. A strategy involving short- or mid-term repeated administrations separated by periods of pause would have the benefit of avoiding immune exacerbation or immune exhaustion. Immune exacerbation can aggravate degenerative processes [112], while immune exhaustion and prolonged resolution processes can leave the body vulnerable to infections or cancer [81,111,113].

There is a somewhat limited number of cytokines that are frequently assessed and reported in IgY research, such as IL-1β, IL-2, IL-6, IL-10, TNF-α, IFN-γ, etc. The results of our experiments showed that many of them had not been significantly modified. This approach, therefore, could lead to missing many immune responses that IgY may in fact induce. The increases in less-reported cytokines caused by the three IgY-containing products investigated in our study, as well as the differences between formulations and treatment times, highlight the necessity of multiplex solutions in the study of immune processes to measure a wider range of cytokines.

## 4. Materials and Methods

### 4.1. Animals

This study used 8-week-old outbred nulliparous C57BL/6 female mice purchased 2 weeks before the start of the experiment from the Bucharest Cantacuzino Institute (Baneasa facility, Bucharest, Romania), with an average weight at the beginning of the experiment of 21.84 g. The animals were housed in standardized, controlled, and sanitary conditions. Mice were acclimated under a 12 h light/dark cycle and identical conditions of temperature (22 ± 0.7 °C) and humidity (60 ± 10%). The mice were housed in autoclavable polycarbonate cages (approximately 300 cm^2^/mouse). The animals had ad libitum access to water (autoclavable bottles with stainless steel sippers) and standardized food (Cantacuzino Institute, Bucharest, Romania) with the following composition: 23% protein, 10% lipids, 50% carbohydrates, 8% crude fiber, 9% vitamin–mineral premix, calcium carbonate and phosphate, and amino acids. A sterilized spruce granulate bedding was used (SAFE^®^ prime S, SAFE^®^, Rosenberg, Germany).

### 4.2. Administered Formulations

The formulations used in the experiment were based on products provided by the SC Romvac SA company (Voluntari, Romania), which are part of their “*Imunoinstant*” line of food supplements: fresh hyperimmune egg, *Imunoinstant G*™, and *Imunoinstant multiplu*™. During the experiment, one group of mice was treated with specific pathogen-free (SPF) eggs, which were also produced by SC Romvac SA.

The hyperimmune eggs were obtained from hens immunized with a mixture of antigens originating from bacterial and fungal pathogens collected from human patients: *Pseudomonas aeruginosa*, *Klebsiella pneumoniae*, *Escherichia coli*, *Enterococcus faecalis*, *Salmonella enteritidis*, *Salmonella typhimurium*, *Streptococcus mutans*, *Staphylococcus aureus*, *Streptococcus group B*, *Proteus mirabilis*, *Acinetobacter baumannii*, *Helicobacter pylori*, *Clostridium difficile* (anatoxin and bacterial cells), *Candida albicans*, *Candida glabrata*, *Candida krusei* [114]. According to the producer, each egg contained, on average, 200 mg of IgY.

*Immunoinstant G*™ is a product obtained by freeze-drying the hyperimmune egg, with a concentration of 6.7 g IgY per 100 g of finished product.

*Imunoinstant multiplu*™ is a product obtained by extracting IgY from the hyperimmune egg yolk. In 80 mL of product, there were 200 mg of IgY in aqueous solution.

### 4.3. Experimental Design

**Figure 7:**
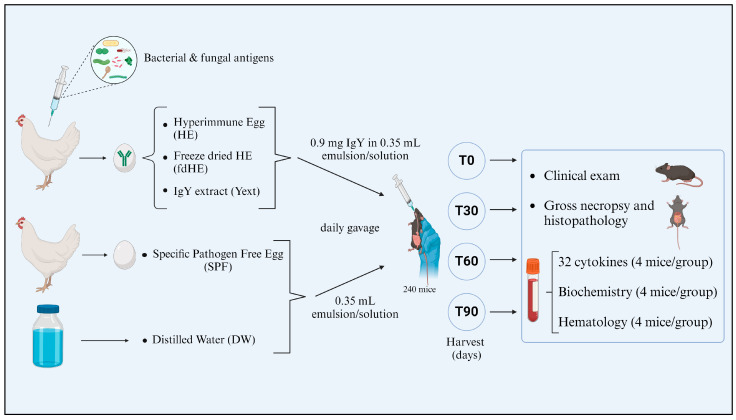
Schematic representation of the experimental design.

A summarized presentation of the experiment is illustrated in Figure 7. A total of 240 C57BL/6 mice were divided into five experimental groups of 48 animals. The experimental groups consisted of 2 control groups and 3 treated groups. One control group received distilled water (DW), while a second control group was given an emulsion of specific pathogen-free egg mixed with distilled water. This second group was used to control for a potential immune response to the normal egg compounds. In all groups, each animal received 0.35 mL of liquid by daily gavage. The treated groups received emulsions of hyperimmune egg (HE) or freeze-dried hyperimmune egg (fdHE) mixed with distilled water, or an aqueous solution of extracted IgY (Yext).

The dose of IgY to be administered to the animals was obtained by converting a relatively high human daily dose of 200 mg IgY (3.33 mg/kg bodyweight) into an equivalent murine dose based on body area (m^2^), according to the formula proposed by Nair and Jacob [115]. The formula used to calculate the dose was:

AED (mg/kg) = HD (mg/kg) × 12.3,

where

AED = Animal Equivalent Dose,
HD = Human Dose
12.3 = Conversion factor for mice.

Based on an HD of 3.33 mg/kg and the average weight of the animals, we used a dose of 41 mg/kg, which corresponded to 0.9 ± 0.03 mg of IgY per mouse. The IgY-containing products were prepared so that the dose was contained in a volume of 0.35 mL of liquid, which was administered daily by gavage. In the case of the SPF group, the emulsion was prepared so that it would contain the same egg amount as the hyperimmune egg emulsion.

At the beginning of the experiment (T0) and at three subsequent time points, namely after 30 days (T30), 60 days (T60), and 90 days (T90) of treatment, 12 mice from each group were harvested to determine the values of a set of hematological (*n* = 4 animals) and biochemical (*n* = 4 animals) parameters and of 32 cytokines (*n* = 4 animals). Blood was obtained by cardiac puncture under anesthesia with 2% isoflurane, then the animals were euthanized with 5% isoflurane. Organs for histopathological examination were also collected.

### 4.4. Histopathology Investigations

For rapid and uniform fixation of organs and tissues, the circulatory system of the mouse was infused with a volume of approximately 20 mL of Lactated Ringer’s solution (for washing the blood of the organs), then with fixing fluid (8% formaldehyde). Mice were subsequently processed for paraffin inclusion. The specimens were embedded in paraffin, sectioned at a 5 μm thickness, and stained with hematoxylin–eosin. The microscopic examination was performed using a Leica DM 750 microscope (Leica Microsystems GmbH, Wetzlar, Germany), and the images were taken with a Leica 5mpx HD camera using the Leica Application Suite (LAS) software, version 4.2.

The histopathological examination included an initial verification of the macroscopic appearance of the tissues and organs compared with the control group, assessing the volume, color, shape, consistency, surface, and appearance of the section surface.

### 4.5. Serum Cytokines Profiles

Kima Vacutest 500 µL containers with separating gel and without anticoagulant were used. After collection, the blood sample was stored at room temperature for 30–60 min, after which it was centrifuged at 5000 rpm for 5 min to separate the serum. The following 32 cytokines were quantified: IL-1α, IL-1β, IL-2, IL-3, IL-4, IL-5, IL-6, IL-9, IL-10, IL-12 (p40), IL-12 (p70), IL-13, IL-17A, Eotaxin, G-CSF, GM-CSF, IFN-γ, KC, MCP-1, MIP-1α, RANTES, TNFα, IL-17F, IL-21, IL-22, IL-23, IL-25 (IL-17E), IL-27, IL-31, IL-33, CD40L, MIP-3α. Serum cytokines were detected through a multiplex ELISA analysis (Bio-Rad Laboratories Inc., Hercules, CA, USA). Bio-Plex Pro Mouse Cytokine 23-plex and Bio-Plex Pro Mouse Th17 Cytokine 10-plex ELISA kits were used by following the protocol of the producer. Data interpretation was performed with the Bio-Plex^®^ MAGPIX™ Multiplex Reader (Bio-Rad Laboratories Inc., Hercules, CA, USA), running Bio-Plex Manager™ MP Software and Bio-Plex Data Pro™ Software 1.2.

### 4.6. Serum Biochemistry Investigations

Blood collection for biochemical investigations was performed in 500 µL vacuum collection tubes with heparin at room temperature, gently homogenizing the tube 5–6 times vertically to prevent clotting. The determination of the biochemical parameters was performed using the IDEXX VetTest Chemistry Analyzer (IDEXX Laboratories Inc., Westbrook, ME, USA). The analyzed parameters were: alkaline phosphatase (ALP), alanine aminotransferase (ALT), blood urea nitrogen (BUN), creatinine (CREAT), glucose (GLU), and total proteins (T-PRO).

### 4.7. Hematology Investigations

Blood collection for hematological investigations was performed in 500 µL vacuum collection tubes with K3 EDTA (Vacutest Kima, Arzergrande, Italy). To prevent clotting, the blood was homogenized with the anticoagulant in the tube by gently rotating the tube vertically 5–6 times. Hematology determinations were performed with the ABAXIS USA vetscan HM2 veterinary hematology analyzer for the following parameters: white blood cells (WBC), lymphocytes (LYM), monocytes (MONO), neutrophils (NEU), eosinophils (EOS), basophils (BAS), red blood cells (RBC), hemoglobin (HGB), hematocrit (HCT), mean corpuscular volume (MCV), mean corpuscular hemoglobin (MCH), mean corpuscular hemoglobin concentration (MCHC), red cell distribution width (RDWs), red cell distribution width % (RDWc), platelets (PLT), plateletcrit (PCT), mean platelet volume (MPV), platelet distribution width (PDWs), and platelet distribution width % (PDWc). 

### 4.8. Statistical Analysis

GraphPad Prism software version 10 (GraphPad Software, Boston, MA, USA) was used for the statistical analysis. The differences between groups and treatment times were assessed by running a two-way ANOVA followed by Tukey’s multiple comparisons test. *p* values (adjusted for multiple comparisons) were considered indicative of statistical significance at values less than 0.05 (*p* < 0.05). In the case of cytokine values reported as “below the detection limit” by the analyzer, the assigned value for statistical analysis was 90% of the LOD.

### 4.9. Ethics

This study was conducted according to the guidelines of Directive 2010/63/EU of the European Parliament on the protection of animals used for scientific purposes and approved by the Ethics Committee of the “Ion Ionescu de la Brad” Iasi University of Life Sciences, decision 524 from 13 April 2020.

## 5. Conclusions

The objective of our study was to assess the effects of the repeated long-term administration of three IgY formulations in non-microbially-challenged C57BL/6 mice by investigating various cytokines and other blood parameters and their correlation with the histopathological aspects.

The results of our research showed that the IgY-containing products were effective at inducing a local and a systemic immune response that varied with formulation and administration time. In the absence of significant amounts of their target antigens in the digestive tract, orally administered IgY appear to act as antigens themselves and trigger an immune response of the GALT by two mechanisms of action: low doses appear to act through a bystander suppression resulting from the induction of oral immunotolerance, while high doses seem to act by initiating an allergic-type pro-inflammatory response, which may then trigger processes of inflammation resolution; these can co-exist with the pro-inflammatory processes, which are maintained by the repeated administration of IgY.

Furthermore, the same dose may have different results in challenge and non-challenge contexts. When administered orally in a microbially non-challenge context, IgYs appear to significantly influence a variety of immunological processes in ways that are not completely understood or easy to predict. High doses may be more effective as antimicrobials in contexts with a microbial challenge, while low doses and intermittent high doses may be better for immunomodulation.

## Figures and Tables

**Table 1:** Cytokines that were significantly increased in the IgY-treated groups compared with both control groups (DW and SPF): the multiplicity-adjusted *p* values of Tukey’s multiple comparison test for the significant differences compared with each of the two control groups and with other sampling times.

	Group	HE	fdHE	Yext
Time				
**T30**		**IL-6:** *p* = 0.0172 (vs. DW) *p* = 0.0296 (vs. SPF) *p* = 0.0177 (vs. T0) *p* = 0.0205 (vs. T60) *p* = 0.0279 (vs. T90) **G-CSF:** *p* < 0.0001 (vs. DW) *p* < 0.0001 (vs. SPF) *p* < 0.0001 (vs. T0) *p* < 0.0001 (vs. T60) *p* = 0.0004 (vs. T90)	**Eotaxin:** *p* = 0.0010 (vs. DW) *p* = 0.0295 (vs. SPF) *p* = 0.0002 (vs. T0) *p* = 0.0436 (vs. T90)	**IL-21:** *p* = 0.0034 (vs. DW) *p* = 0.0016 (vs. SPF) *p* = 0.0010 (vs. T0) *p* = 0.0010 (vs. T60) *p* = 0.0018 (vs. T90) **IL-25 (IL-17E):** *p* = 0.0064 (vs. DW) *p* = 0.0054 (vs. SPF) *p* = 0.0298 (vs. T60) *p* = 0.0127 (vs. T90) **IL-27:** *p* = 0.0226 (vs. DW) *p* = 0.0124 (vs. SPF) *p* = 0.0058 (vs. T0) *p* = 0.0058 (vs. T60) *p* = 0.0198 (vs. T90) **CD40L:** *p* < 0.0001 (vs. DW) *p* < 0.0001 (vs. SPF) *p* < 0.0001 (vs. T0) *p* < 0.0001 (vs. T60) *p* < 0.0001 (vs. T90)
**T60**		**IL-31:** *p* = 0.0351 (vs. DW) *p* = 0.0351 (vs. SPF) *p* = 0.0378 (vs. T0) *p =* 0.0374 (vs. T30)	–	–
**T90**		**IL-2:** *p* < 0.0001 (vs. DW) *p* < 0.0001 (vs. SPF) *p* = 0.0002 (vs. T0) *p* = 0.0206 (vs. T30) **IL-13:** *p* = 0.0001 (vs. DW) *p* = 0.0370 (vs. SPF) *p* = 0.0087 (vs. T0) *p* = 0.0177 (vs. T60) **G-CSF:** *p* = 0.0101 (vs. DW) *p* = 0.0005 (vs. SPF) *p* = 0.0012 (vs. T0) *p* = 0.0004 (vs. T30) *p* = 0.0005 (vs. T60) **KC (CXCL1):** *p* < 0.0001 (vs. DW) *p* < 0.0001 (vs. SPF) *p* < 0.0001 (vs. T0) *p* < 0.0001 (vs. T30) *p* < 0.0001 (vs. T60)	**IL-5:** *p* = 0.0002 (vs. DW) *p* = 0.0338 (vs. SPF) *p* = 0.0006 (vs. T0) *p* = 0.0134 (vs. T30) *p* = 0.0019 (vs. T60) **IL-10:** *p* < 0.0001 (vs. DW) *p* = 0.0115 (vs. SPF) *p* = 0.0006 (vs. T0) *p* = 0.0381 (vs. T60) **IL-12 (p40):** *p* < 0.0001 (vs. DW) *p* = 0.0001 (vs. SPF) *p* < 0.0001 (vs. T0) *p* = 0.0003 (vs. T60) **TNFα:** *p* < 0.0001 (vs. DW) *p* = 0.0276 (vs. SPF) *p* < 0.0001 (vs. T0) *p* = 0.0002 (vs. T30) *p* = 0.0117 (vs. T60)	**MIP-3α:** *p* < 0.0001 (vs. DW) *p* < 0.0001 (vs. SPF) *p* < 0.0001 (vs. T0) *p* < 0.0001 (vs. T30) *p* < 0.0001 (vs. T60)

**Table 2:** Cytokines that were increased in the IgY-treated groups compared with both control groups (DW and SPF): the type of immune response in which they are reported to play an active role, with references.

	Group	HE	fdHE	Yext
Time				
**T30**		**IL-6**: Th2 *, Th17 [27,28,29,30] **G-CSF**: Th17 [31]	**Eotaxin**: Th2, Th17 [28,32]	**IL-21**: Th17 [30,33] **IL-25 (IL-17E)**: Th2 [30,34] **IL-27**: Th1, Th2 [35] **CD40L**: Th1, Th2, Th17 [36]
**T60**		**IL-31**: Th2 [37]	–	–
**T90**		**IL-2**: Th1, Th2 [27,38] **IL-13**: Th2 [27,28] **G-CSF**: Th17 [31] **KC (CXCL1)**: Th17 [39]	**IL-5**: Th2 [40,41] **IL-10**: Th2 [27] **IL-12 (p40)**: Th1, Th17 [42] **TNFα**: Th1, Th2, Th17 [27,30,43]	**MIP-3α**: Th17 [44]

* The colors mark the different types of immune response: Th1 type (black), Th2 type (red), Th17 type (blue).

**Table 3:** The values (mean ± SD) of hematological parameters associated to RBCs and platelets: at the beginning of the experiment (T0) and at the three harvesting time points of 30 days (T30), 60 days (T60), 90 days (T90).

Time	Group	RBC (10^12^/L)	HGB (g/dL)	HCT (%)	MCV (fl)	MCH (pg)	MCHC (g/dL)	RDWc (%)	RDWs (%)	PLT (10^9^/L)	PCT (%)	MPV (fl)	PDWc (%)	PDWs (fl)
T0	DW	9.56 ±0.12	13.20 ±0.76	40.92 ±1.91	41.15 ±0.56	13.13 ±0.71	31.12 ±0.23	19.53 ±0.16	31.12 ±0.88	283.00 ±43.10	0.15 ±0.78	6.01 ±0.18	27.00 ±0.22	6.67 ±0.22
	SPF	9.72 ±1.16	14.12 ±0.32	42.38 ±0.89	42.11 ±0.62	13.23 ±0.43	32.14 ±0.56	19.43 ±0.63	30.93 ±0.10	291.00 ±32.10	0.16 ±0.03	6.00 ±0.11	28.01 ±0.21	7.00 ±0.08
	HE	9.43 ±1.34	12.54 ±0.14	41.08 ±1.76	41.31 ±0.78	13.00 ±0.83	31.44 ±0.72	19.45 ±0.73	31.33 ±0.12	287.00 ±42.00	0.15 ±0.39	6.00 ±0.22	27.08 ±0.14	6.80 ±0.03
	fdHE	9.62 ±1.34	13.48 ±0.19	42.62 ±0.86	42.00 ±0.18	13.08 ±0.61	31.83 ±0.64	19.13 ±0.13	30.33 ±0.87	286.00 ±12.00	0.15 ±0.39	6.00 ±0.12	27.43 ±0.19	6.56 ±0.18
	Yext	9.71 ±1.34	12.48 ±0.19	43.28 ±0.86	42.00 ±0.35	13.33 ±0.41	31.52 ±0.56	19.51 ±0.11	31.21 ±0.24	293.00 ±12.00	0.15 ±0.32	6.20 ±0.18	27.23 ±0.19	6.57 ±0.24
T30	DW	9.69 ±0.74	13.22 ±0.59	42.97 ±0.70	42.75 ±0.49	13.55 ±0.26	31.75 ±0.45	19.50 ±0.44	31.99 ±0.88	286.00 ±58.40	0.16 ±0.03	6.17 ±0.14	27.00 ±0.49	6.82 ±0.24
	SPF	9.96 ±1.37	14.15 ±0.15	43.21 ±1.64	42.05 ±0.71	13.37 ±0.12	32.10 ±0.48	19.50 ±0.43	30.67 ±0.30	347.00 ±85.50	0.20 ±0.04	6.05 ±0.08	28.70 ±0.60	7.70 ±0.34
	HE	9.84 ±0.48	12.97 ±0.51	40.08 ±1.85	41.75 ±0.82	13.17 ±0.28	31.77 ±0.32	19.25 ±0.28	30.03 ±0.34	354.00 ±29.40	0.21 ±0.01	6.17 ±0.19	28.15 ±0.62	7.37 ±0.86
	fdHE	10.27 ±0.54	14.17 ±0.39	43.36 ±1.36	42.25 ±0.82	13.85 ±0.45	32.77 ±0.54	19.55 ±0.57	31.05 ±0.61	412.00 ±32.00	0.27 ±0.09	6.70 ±0.21	28.95 ±1.01	7.87 ±0.29
	Yext	10.49 ±0.55	14.40 ±0.53	44.17 ±2.26	42.25 ±0.43	13.70 ±0.25	32.07 ±0.57	19.67 ±0.83	31.45 ±1.21	349.00 ±66.50	0.21 ±0.03	6.12 ±0.17	29.47 ±2.53	8.27 ±1.70
T60	DW	9.81 ±0.42	13.52 ±0.36	43.24 ±0.97	43.25 ±0.82	13.45 ±0.26	31.20 ±0.33	18.82 ±0.43	30.87 ±0.64	417.50 ±52.47	0.26 ±0.05	6.30 ±0.24	28.00 ±0.45	7.20 ±0.24
	SPF	10.51 ±0.49	14.20 ±0.45	44.50 ±1.69	42.50 ±0.50	13.52 ±0.29	31.85 ±0.55	19.25 ±0.61	30.73 ±0.91	440.00 ±24.48	0.27 ±0.02	6.15 ±0.21	28.40 ±0.45	7.20 ±0.24
	HE	10.30 ±0.42	14.00 ±0.36	44.56 ±1.26	43.25 ±1.20	13.52 ±0.34	31.45 ±0.26	19.27 ±0.41	31.11 ±0.69	399.00 ±43.03	0.24 ±0.04	5.90 ±0.10	28.07 ±1.75	7.30 ±1.02
	fdHE	10.35 ±0.60	13.67 ±0.75	43.12 ±1.94	43.00 ±0.70	13.60 ±0.20	31.72 ±0.49	20.75 ±1.05	33.77 ±2.45	361.25 ±41.44	0.24 ±0.08	6.10 ±0.12	28.80 ±1.06	7.77 ±0.65
	Yext	10.45 ±0.40	13.25 ±0.37	41.95 ±0.89	43.00 ±1.22	13.55 ±0.18	31.50 ±0.67	19.00 ±0.52	30.85 ±0.35	434.00 ±55.21	0.27 ±0.03	6.20 ±0.27	28.80 ±1.40	7.77 ±0.84
T90	DW	9.21 ±0.50	13.20 ±0.64	37.38 ±2.58	40.50 ±1.11	14.35 ±0.22	35.35 ±0.83	18.10 ±0.33	27.70 ±0.40	236.75 ±10.25	0.16 ±0.07	6.55 ±0.61	32.57 ±3.09	10.50 ±2.37
	SPF	10.55 ±0.30	15.25 ±0.45	41.34 ±1.21	39.25 ±1.29	14.45 ±0.30	37.07 ±1.23	19.00 ±0.27	28.30 ±0.87	471.25 ±27.18	0.29 ±0.16	6.17 ±0.08	28.82 ±0.78	2.77 ±0.48
	HE	10.42 ±0.48	14.77 ±0.87	42.01 ±1.95	40.00 ±1.34	14.17 ±0.25	35.12 ±0.55	18.87 ±0.44	28.70 ±0.66	351.00 ±16.34	0.21 ±0.09	6.27 ±0.19	30.52 ±1.58	8.87 ±1.04
	fdHE	10.62 ±0.39	14.85 ±0.45	42.94 ±1.13	40.25 ±0.43	14.17 ±0.21	34.57 ±0.59	18.87 ±0.40	28.70 ±0.34	313.00 ±10.53	0.20 ±0.05	6.47 ±0.30	31.72 ±1.94	9.67 ±1.27
	Yext	10.45 ±0.11	14.45 ±0.29	42.36 ±0.93	40.05 ±0.50	13.85 ±0.16	34.12 ±0.19	18.35 ±0.51	27.90 ±0.83	466.75 ±38.27	0.29 ±0.02	6.29 ±0.02	29.45 ±1.32	8.15 ±0.81

## Data Availability

Data are contained within the article or Supplementary Material.

## Supplementary Materials

The following supporting information can be downloaded at: https://www.mdpi.com/article/10.3390/ijms25168701/s1.
