# Supplementary material for: Long-Term Oral Administration of Hyperimmune Egg-Based IgY-Rich Formulations Induces Mucosal Immune Response and Systemic Increases of Cytokines Involved in Th2- and Th17-Type Immune Responses in C57BL/6 Mice
Source: Int J Mol Sci. 2024 Aug 9;25(16):8701. doi: 10.3390/ijms25168701 (PMC11354499; doi:10.3390/ijms25168701)
Supplement: Supplementary file 1 [file ijms-25-08701-s001.zip › Table-S1-Bodyweight.pdf]

Table S1. Bodyweight (Mean ± SD) at the beginning of the experiment (T0) and at the three harvesting time points of 30 days (T30), 60 days (T60), 90 days (T90).

	DW	SPF	HE	fdHE	Yext
**T0**	22.05 ± 0.43	21.52 ± 0.68	21.75 ± 0.62	21.55 ± 1.21	22.35 ± 0.67
**T30**	22.06 ± 0.17	20.74 ± 0.39	21.04 ± 0.48	20.15 ± 0.52	21.94 ± 0.23
**T60**	23.3 ± 0.32	22.03 ± 0.14	21.97 ± 0.33	22.12 ± 0.41	21.99 ± 0.37
**T90**	23.18 ± 0.24	21.79 ± 0.22	22.24 ± 0.11	22.38 ± 0.62	23.14 ± 0.12
