# Supplementary material for: Long-Term Oral Administration of Hyperimmune Egg-Based IgY-Rich Formulations Induces Mucosal Immune Response and Systemic Increases of Cytokines Involved in Th2- and Th17-Type Immune Responses in C57BL/6 Mice
Source: Int J Mol Sci. 2024 Aug 9;25(16):8701. doi: 10.3390/ijms25168701 (PMC11354499; doi:10.3390/ijms25168701)
Supplement: Supplementary file 1 [file ijms-25-08701-s001.zip › Table-S2-serum-cytokines.pdf]

Table S2. The values of serum cytokines (Mean ± SD): at the beginning of the experiment (T0) and at the three harvesting time points of 30 days (T30), 60 days (T60), 90 days (T90).

Time	Group	CITOKINES (pg/ml)											
		**IL-1α**	**IL-1β**	**IL-2**	**IL-3**	**IL-4**	**IL-5**	**IL6**	**IL-9**	**IL-10**	**IL-12 (p40)**	**IL-12 (p70)**	**IL-13**
**T0**	**DW**	7.79±1.17	7.8±1.32	**0.54	1.77±2.0	0.79±0.12	4.02±3.11	2.21±0.62	2.21±3.45	16.81±5.0	391.10±6.76	56.0±0.13	34.53±1.21
	**SPF**	7.56±0.18	7.1±1.1	4.23±0.36	1.63±1.89	0.63±0.19	4.12±1.67	1.96±1.02	2.63±2.65	16.87±3.0	386.15±24.0	55.03±0.88	32.03±0.16
	**HE**	6.98±1.23	6.3±0.19	4.01±0.45	1.45±2.04	0.69±0.02	4.32±2.61	2.24±0.35	2.83±1.83	16.65±5.0	334.6±15.12	56.08±0.73	33.78±1.34
	**fdHE**	7.22±0.45	**8.46	*4.39	1.18±1.32	0.71±0.32	4.43±1.98	2.34±0.13	2.15±3.34	16.98±3.05	392.44±31.0	55.87±0.23	33.98±0.61
	**Yext**	7.0±1.34	**8.46	3.73±1.43	1.67±2.14	0.74±1.03	4.08±1.32	2.02±0.69	2.79±3.30	15.87±2.35	326.00±0.22	56.14±0.66	34.13±1.45
**T30**	**DW**	5.38±1.71	*1.26	4.91±0.48	2.15±0.79	0.9±0.61	6.93±1.65	3.17±0.98	17.8±5.86	25.37±10.9	269.02±14.85	62.7±14.03	36.24±16.0
	**SPF**	6.17±1.98	**8.46	4.13±0.82	2.71±0.64	1.29±0.83	9.6±3.01	3.99±1.12	19.86±3.84	22.87±5.12	230.0±17.06	60.95±18.91	45.03±8.10
	**HE**	6.19±1.42	*1.06	6.77±2.8	2.72±0.52	1.82±0.67	15.22±9.4	16.36±25.64	16.72±6.13	25.38±5.5	229.94±18.77	60.54±10.36	40.98±17.64
	**fdHE**	5.00±0.33	**8.46	3.39±0.63	1.74±1.52	0.68±0.37	7.2±1.52	2.76±0.30	12.67±1.39	27.81±2.23	297.22±39.36	44.46±20.55	*34.53
	**Yext**	4.24±0.42	**8.46	**0.54	4.89±0.81	1.05±0.39	4.89±0.81	2.64±0.04	15.02±1.95	24.08±1.28	208.85±17.93	65.39±4.07	29.85±4.04
**T60**	**DW**	4.52±0.30	**8.46	2.32±0.35	2.04±0.54	1.09±1.09	5.49±1.41	2.88±0.75	15.69±4.9	20.02±3.54	263.65±47.62	60.28±10.66	28.54±11.53
	**SPF**	5.01±1.48	**8.46	3.21±1.07	1.97±0.56	0.80±0.46	4.89±0.90	2.29±0.17	12.85±2.55	25.02±4.09	243.66±28.72	55.82±9.13	35.36±18.33
	**HE**	5.99±2.98	**8.46	8.08±3.0	1.91±0.12	0.78±0.17	3.47±1.00	3.52±0.88	13.54±2.86	20.61±3.55	221.41±28.69	50.52±5.52	36.47±3.36
	**fdHE**	3.92±0.94	**8.46	3.21±1.31	2.13±0.32	0.79±0.9	5.43±0.46	2.61±0.27	16.28±1.82	23.80±1.46	213.40±44.32	59.16±3.82	36.08±7.41
	**Yext**	3.53±0.44	**8.46	2.67±1.08	1.57±0.54	0.95±0.4	3.68±1.43	2.20±0.7	11.26±5.47	17.38±0.52	233.74±12.47	45.72±12.94	*27.52
**T90**	**DW**	2.98±0.30	**8.46	**0.54	1.25±0.28	**1.89	3.25±0.40	2.11±0.18	9.66±0.64	16.44±2.32	176.34±29.48	40.65±5.32	*17.76
	**SPF**	3.71±0.29	1.56±2.26	3.21±1.23	2.4±1.05	0.9±0.34	7.69±5.11	2.68±0.88	14.26±7.37	22.0±5.33	206.21±24.3	56.95±18.29	37.68±10.16
	**HE**	3.93±0.80	**8.46	12.47±10.92	1.71±0.72	0.85±0.24	5.41±0.95	4.02±2.85	15.13±2.30	22.77±3.4	223.78±4.02	47.74±12.74	68.81±56.68
	**fdHE**	3.81±0.84	4.40±0.43	3.91±0.44	3.71±1.60	1.33±0.20	15.83±11.83	4.07±0.16	19.50±1.06	31.96±2.08	292.05±21.75	68.36±8.2	42.06±2.95
	**Yext**	2.61±0.39	**8.46	**0.54	0.93±0.34	**1.89	3.25±0.40	1.90±0.38	*7.49	15.53±1.43	178.24±23.67	37.68±1.34	**34.83

Table S4 (continued)

Time	Group	CITOKINES (pg/ml)										
		IL-17A	EOTAXIN	G-CSF	GM-CSF	IFN-γ	KC (CXCL1)	MCP-1	MIP-1α	RANTES	TNFα	IL-17F
T0	DW	4.71±1.02	319.12±96.77	42.7±2.10	22.2±3.53	10.78±1.8	14.2±4.8	211.62±10.65	2.01±0.67	46.17±4.32	30.48±8.12	0.23±0.13
	SPF	4.34±1.23	337.44±53.34	43.3±2.23	22.8±2.43	10.89±0.6	14.8±3.15	193.52±11.33	2.18±0.67	45.78±3.56	31.12±4.43	0.20±0.18
	HE	4.84±1.56	343.87±66.17	43.8±1.15	23.1±1.19	9.82±2.8	14.62±3.4	213.94±10.18	2.26±0.67	45.96±3.87	30.63±3.19	0.20±0.33
	fdHE	3.68±1.86	369.18±38.32	42.3±2.78	22.4±2.58	9.54±2.3	13.78±4.3	216.23±9.44	2.09±0.67	46.33±3.42	29.67±7.03	0.21±0.18
	Yext	4.23±1.08	356.82±45.36	43.7±3.21	22.0±1.81	10.18±0.8	14.24±3.93	201.82±10.37	2.04±0.67	45.19±4.81	30.32±6.45	0.23±0.25
T30	DW	10.92±3.94	421.65±67.51	44.98±3.35	21.48±4.8	10.6±2.78	20.61±4.54	104.05±22.54	2.90±0.76	51.88±12.34	37.38±7.65	0.38±0.06
	SPF	19.56±12.42	603.98±102.40	47.50±1.67	26.91±3.63	12.79±2.93	23.82±3.41	112.58±0.13	3.37±0.13	54.37±14.43	44.99±9.89	0.12±0.09
	HE	15.96±4.69	725.39±208.11	117.14±19.03	26.32±5.15	11.24±3.37	24.02±6.47	112.21±16.15	3.34±0.49	48.30±9.32	41.87±9.67	0.18±0.15
	fdHE	10.64±5.93	1092±761.59	46.53±10.19	19.46±3.89	8.13±0.98	20.33±4.36	123.22±59.97	2.78±0.23	51.47±18.54	31.68±5.88	0.17±0.15
	Yext	28.0±4.29	894.02±165.50	51.92±14.55	23.0±3.55	10.67±1.02	16.97±1.41	95.05±9.19	2.89±0.20	43.33±4.76	40.92±3.30	1.08±1.57
T60	DW	19.23±3.82	429.72±182.21	48.17±18.03	23.27±3.98	10.25±2.96	20.25±1.39	103.27±14.95	3.88±0.58	53.46±5.52	37.39±7.76	0.25±0.08
	SPF	15.96±6.52	522.17±45.03	37.57±7.81	19.75±2.15	8.96±1.44	21.29±12.09	108.56±25.62	2.77±0.31	50.37±22.06	36.62±6.58	0.07±0.05
	HE	13.74±7.83	468.47±169.16	41.11±16.16	20.19±4.50	9.93±1.34	19.63±2.39	92.22±5.58	3.40±1.32	55.47±11.09	33.44±4.54	0.03±0.01
	fdHE	22.18±5.05	846.2±316.47	42.81±11.96	22.69±1.94	10.33±1.19	18.64±0.31	104.33±17.66	3.96±0.24	72.71±13.57	38.72±1.93	0.12±0.09
	Yext	14.32±6.96	435.6±29.27	39.87±16.70	17.43±4.17	8.32±2.39	14.42±0.38	108.82±30.50	2.48±0.57	56.79±12.11	31.4±8.27	0.21±0.11
T90	DW	19.46±10.73	463.60±199.20	48.54±10.11	14.17±1.46	6.13±1.18	15.22±1.49	75.87±8.48	2.05±0.52	23.91±7.03	26.52±4.01	0.36±0.40
	SPF	24.25±9.86	858.92±247.55	40.26±14.86	22.61±7.77	10.32±3.88	20.74±5.79	95.99±25.81	2.8±0.79	37.62±6.30	39.54±15.29	0.04±0.12
	HE	21.64±0.49	527.84±141.98	79.07±23.6	28.03±7.8	10.53±1.39	48.34±3.80	84.83±31.11	2.35±0.86	31.33±1.47	35.69±7.00	0.07±0.05
	fdHE	35.34±5.89	655.67±194.14	57.13±22.30	25.75±0.97	13.48±1.02	22.98±1.71	115.60±22.59	3.22±0.45	39.82±9.15	56.15±12.62	0.12±0.09
	Yext	10.57±4.11	601.52±189.91	37.46±7.24	11.44±3.09	5.33±0.88	14.29±1.65	67.13±17.34	3.03±1.40	29.95±9.56	25.60±7.46	0.36±0.19

Table S4 (continued)

Time	Group	CITOKINES (pg/ml)								
		IL-21	IL-22	IL-23	IL-25 (IL-17E)	IL-27	IL-31	IL-33	CD40L	MIP-3α
T0	DW	**2.52	3.3±1.89	**0.63	3.19±0.23	0.44±0.05	8.58±4.32	8.34±3.0	**0.36	0.69±1.32
	SPF	**2.52	2.9±1.43	**0.63	3.06±0.14	0.45±0.18	9.03±2.32	8.65±3.08	**0.36	0.71±1.18
	HE	**2.52	3.4±0.34	**0.63	2.89±0.45	0.43±0.45	8.67±3.83	9.66±2.12	**0.36	0.73±1.44
	fdHE	**2.52	2.9±1.96	**0.63	2.63±0.30	0.44±0.08	8.22±4.21	8.12±3.43	**0.36	0.69±2.08
	Yext	**2.52	3.0±1.32	**0.63	3.08±0.18	0.43±0.24	9.23±1.68	7.94±3.23	**0.36	0.70±1.42
T30	DW	17.92±31.01	2.54±1.77	8.73±7.88	0.54±0.36	*1.18	35.90±23.66	29.88±5.75	**0.36	1.03±0.34
	SPF	**2.52	1.21±0.63	1.32±0.91	0.41±0.28	0.68±0.42	27.53±19.08	14.39±8.44	**0.36	*1.04
	HE	**2.52	3.11±2.96	**0.63	0.79±0.69	0.77±0.45	8.58±12.05	32.71±14.36	**0.36	3.23±4.10
	fdHE	**2.52	0.86±0.56	4.96±2.24	1.04±0.79	0.72±0.39	17.02±14.61	19.75±13.68	**0.36	0.80±0.20
	Yext	268.47±419.28	1.54±0.73	26.11±39.93	8.77±13.22	8.4±13.79	76.41±16.51	40.18±47.74	158.15±0.5	3.26±3.25
T60	DW	**2.52	1.43±1.17	**0.63	0.75±0.13	*0.44	**4.68	**0.18	**0.36	1.81±0.49
	SPF	**2.52	0.77±0.36	*2.38	1.00±0.43	0.25±0.16	**4.68	13.11±1.08	**0.36	2.39±3.25
	HE	14.26±9.88	1.61±1.66	*2.38	1.47±1.56	0.93±0.42	64.59±97.01	**0.18	**0.36	1.37±0.57
	fdHE	14.26±9.88	2.51±1.16	**0.63	0.79±0.69	*0.44	30.5±52.82	**0.18	**0.36	0.91±0.71
	Yext	**2.52	1.00±0.27	*2.38	2.18±2.47	*0.44	7.62±1.65	8.34±2.05	**0.36	1.26±0.52
T90	DW	**2.52	3.02±0.94	*2.38	2.91±1.02	2.91±1.02	7.62±1.65	**0.18	**0.36	**0.27
	SPF	**2.52	4.68±3.04	**0.63	0.10±0.02	*0.12	**4.68	**0.18	**0.36	0.69±0.35
	HE	14.26±9.88	2.44±1.10	**0.63	5.13±2.85	5.13±2.85	22.24±23.66	**0.18	**0.36	*0.69
	fdHE	**2.52	1.18±0.64	**0.63	3.21±4.2	3.21±4.22	**4.68	**0.18	**0.36	0.57±0.20
	Yext	14.26±9.88	2.52±1.50	*2.38	1.45±1.30	1.45±1.31	47.02±45.80	**0.18	**0.36	36.62±25.75

* = Value extrapolated beyond standard range

** = 90% of the Limit of Detection value
