# Supplementary material for: Long-Term Oral Administration of Hyperimmune Egg-Based IgY-Rich Formulations Induces Mucosal Immune Response and Systemic Increases of Cytokines Involved in Th2- and Th17-Type Immune Responses in C57BL/6 Mice
Source: Int J Mol Sci. 2024 Aug 9;25(16):8701. doi: 10.3390/ijms25168701 (PMC11354499; doi:10.3390/ijms25168701)
Supplement: Supplementary file 1 [file ijms-25-08701-s001.zip › Table-S3-Biochemistry.pdf]

Table S3. The values of serum biochemistry parameters (Mean ± SD): alkaline phosphatase (ALP), alanine aminotransferase (ALT), glucose (GLU), total proteins (T-PRO), blood urea nitrogen (BUN), and creatinine (CREAT): at the beginning of the experiment (T0) and at the three harvesting time points of 30 days (T30), 60 days (T60), 90 days (T90).

Time	Group	BUN (mg/dl)	GLU (mg/dl)	ALP (U/L)	T-PRO (g/dl)	ALT (U/L)	CREAT (mg/dl)
T0	DW	23.00 ± 1.67	97.60 ± 0.30	145.50 ± 1.42	5.00 ± 0.21	21.20 ± 1.63	0.71 ± 0.01
	SPF	20.00 ± 1.22	82.30 ± 2.28	162.50 ± 2.70	5.05 ± 0.14	21.30 ± 1.05	0.60 ± 0.30
	HE	23.14 ± 0.20	97.50 ± 1.50	164.70 ± 1.50	5.34 ± 0.90	22.60 ± 2.02	0.50 ± 0.12
	fdHE	23.50 ± 2.16	93.00 ± 2.04	160.00 ± 3.00	5.60 ± 0.30	23.00 ± 1.60	0.60 ± 0.08
	Yext	22.34 ± 1.02	99.20 ± 1.21	165.00 ± 5.00	5.35 ± 0.50	23.50 ± 1.50	0.75 ± 0.13
T30	DW	23.50 ± 6.36	99.50 ± 40.30	155.50 ± 1.71	5.30 ± 0.14	21.50 ± 3.53	0.75 ± 0.07
	SPF	20.00 ± 2.82	83.00 ± 32.34	163.50 ± 9.50	5.25 ± 0.25	21.50 ± 0.50	0.60 ± 1.02
	HE	30.00 ± 0.20	157.50 ± 1.50	170.50 ± 2.50	6.10 ± 0.70	30.00 ± 2.00	0.50 ± 0.32
	fdHE	26.50 ± 6.36	183.00 ± 15.00	210.00 ± 9.00	5.90 ± 0.10	54.00 ± 3.80	0.90 ± 0.10
	Yext	30.00 ± 1.41	180.00 ± 13.00	225.00 ± 25.00	6.35 ± 0.35	26.50 ± 2.50	0.85 ± 0.05
T60	DW	43.50 ± 4.50	96.50 ± 4.50	179.50 ± 6.50	5.45 ± 0.05	65.00 ± 0.50	0.90 ± 0.00
	SPF	29.00 ± 0.11	104.50 ± 1.50	150.00 ± 5.00	5.35 ± 0.15	71.50 ± 5.50	0.95 ± 0.05
	HE	26.00 ± 1.00	147.00 ± 7.00	154.50 ± 2.00	5.50 ± 0.30	26.00 ± 16.00	0.95 ± 0.05
	fdHE	21.00 ± 2.20	168.00 ± 3.50	237.00 ± 39.00	6.90 ± 0.80	271.50 ± 14.50	0.85 ± 0.15
	Yext	32.00 ± 1.00	154.00 ± 2.00	211.00 ± 35.00	5.90 ± 0.10	177.00 ± 67.00	0.80 ± 0.30
T90	DW	33.30 ± 4.49	95.00 ± 12.02	155.33 ± 2.88	5.46 ± 0.09	17.33 ± 4.78	0.56 ± 0.09
	SPF	26.00 ± 3.55	138.66 ± 34.10	113.00 ± 8.48	5.10 ± 0.43	10.50 ± 6.17	0.76 ± 0.04
	HE	18.00 ± 2.16	142.00 ± 8.65	113.80 ± 13.63	5.50 ± 0.08	43.66 ± 37.93	0.53 ± 0.16
	fdHE	20.33 ± 0.94	137.00 ± 12.03	163.30 ± 53.95	5.25 ± 0.02	41.00 ± 20.31	0.46 ± 0.04
	Yext	32.66 ± 2.86	103.00 ± 18.45	116.00 ± 19.79	5.70 ± 0.21	10.50 ± 11.73	0.40 ± 0.08
