# Supplementary material for: Long-Term Oral Administration of Hyperimmune Egg-Based IgY-Rich Formulations Induces Mucosal Immune Response and Systemic Increases of Cytokines Involved in Th2- and Th17-Type Immune Responses in C57BL/6 Mice
Source: Int J Mol Sci. 2024 Aug 9;25(16):8701. doi: 10.3390/ijms25168701 (PMC11354499; doi:10.3390/ijms25168701)
Supplement: Supplementary file 1 [file ijms-25-08701-s001.zip › Table-S4-Hematology-WBC.pdf]

Table S4. Total WBC levels and the values of of neutrophils (NEU), monocytes (MONO) and lymphocytes (LYM) in absolute numbers (109/L) and percentages of total white blood cells (Mean ± SD): at the beginning of the experiment (T0) and at the three harvesting time points of 30 days (T30), 60 days (T60), 90 days (T90).

Time	Group	WBC (10*9/l)	NEU (10*9/l)	LYM (10*9/l)	MONO (10*9/l)	EOS (10*9/l)	BASO (10*9/l)	NEU (%)	LYM (%)	MONO (%)	EOS (%)	BASO (%)
	DW	1.96 ±0.22	0.13 ±0.13	1.42 ±0.43	0.05 ±0.01	< DL	< DL	6.00 ±1.88	89.34 ±1.87	3.60 ±1.78	< DL	< DL
	SPF	1.65 ±0.34	0.12 ±0.21	1.33 ±0.87	0.04 ±0.01	< DL	< DL	6.51 ±1.67	81.40 ±1.38	2.62 ±1.02	< DL	< DL
T0	HE	1.76 ±0.95	0.14 ±0.11	1.40 ±0.26	0.05 ±0.02	< DL	< DL	7.40 ±1.32	70.21 ±1.29	3.23 ±1.08	< DL	< DL
	fdHE	1.98 ±0.73	0.12 ±0.37	1.39 ±0.03	0.04 ±0.02	< DL	< DL	7.11 ±0.32	77.46 ±1.93	3.50 ±2.00	< DL	< DL
	Yext	1.84 ±0.23	0.14 ±0.71	1.36 ±0.98	0.05 ±0.07	< DL	< DL	7.50 ±1.25	80.23 ±1.18	2.30 ±0.76	< DL	< DL
	DW	1.97 ±0.47	0.12 ±0.02	1.44 ±0.56	0.05 ±0.00	< DL	< DL	6.02 ±2.38	89.87 ±2.37	3.90 ±1.01	< DL	< DL
	SPF	1.62 ±0.56	0.19 ±0.08	1.24 ±0.74	0.04 ±0.03	< DL	< DL	16.65 ±17.59	90.60 ±17.18	2.75 ±1.04	< DL	< DL
T30	HE	5.18 ±1.15	1.03 ±1.11	1.90 ±1.14	1.10 ±1.61	< DL	< DL	23.70 ±11.89	70.47 ±12.34	5.72 ±2.05	< DL	< DL
	fdHE	7.19 ±3.01	0.91 ±0.57	4.73 ±3.12	0.43 ±0.52	< DL	< DL	15.72 ±1.52	78.82 ±2.96	5.50 ±4.50	< DL	< DL
	Yext	6.93 ±1.45	0.94 ±0.92	4.27 ±2.55	0.06 ±0.03	< DL	< DL	13.70 ±7.55	83.97 ±5.92	2.32 ±1.80	< DL	< DL
	DW	2.41 ±1.25	0.12 ±0.13	2.22 ±1.12	0.07 ±0.04	< DL	< DL	4.60 ±3.67	92.50 ±3.14	2.82 ±1.05	< DL	< DL
	SPF	3.40 ±2.21	0.26 ±0.33	3.08 ±2.18	0.05 ±0.01	< DL	< DL	7.40 ±9.30	90.30 ±9.64	2.27 ±1.01	< DL	< DL
T60	HE	5.36 ±2.39	0.94 ±0.74	4.20 ±1.86	0.22 ±0.10	< DL	< DL	16.10 ±8.20	79.90 ±8.98	3.90 ±0.75	< DL	< DL
	fdHE	4.51 ±1.88	0.47 ±0.66	3.83 ±2.18	0.08 ±0.06	< DL	< DL	12.02 ±5.40	79.90 ±8.98	4.05 ±3.00	< DL	< DL
	Yext	7.47 ±3.78	0.44 ±0.45	5.60 ±3.78	0.51 ±0.40	< DL	< DL	6.40 ±3.90	89.07 ±5.91	4.65 ±3.72	< DL	< DL
	DW	2.40 ±2.05	0.29 ±0.04	2.60 ±1.22	0.54 ±0.33	< DL	< DL	7.12 ±6.94	76.97 ±5.94	3.90 ±3.20	< DL	< DL
	SPF	3.83 ±2.66	0.57 ±0.64	4.14 ±2.24	0.11 ±0.04	< DL	< DL	10.22 ±6.07	86.37 ±4.95	3.40 ±2.12	< DL	< DL
T90	HE	4.36 ±2.74	0.42 ±0.45	3.82 ±2.22	0.19 ±0.13	< DL	< DL	7.20 ±5.93	90.30 ±5.50	2.60 ±1.35	< DL	< DL
	fdHE	4.37 ±1.97	0.18 ±0.10	3.84 ±2.00	0.15 ±0.10	< DL	< DL	4.55 ±2.94	91.05 ±6.10	5.22 ±3.42	< DL	< DL
	Yext	3.63 ±0.55	1.40 ±0.58	6.45 ±1.59	0.12 ±0.08	< DL	< DL	11.82 ±5.69	83.37 ±5.54	3.77 ±1.76	< DL	< DL

< DL = below the detection limit
